# Type 2 Diabetes: Hypoinsulinism, Hyperinsulinism, or Both?

**DOI:** 10.1371/journal.pmed.0040148

**Published:** 2007-04-03

**Authors:** Benjamin Glaser

## Abstract

The author discusses a new study reporting the birth weight of patients carrying a mutation in either of two closely related genes associated with maturity-onset diabetes of the young, testing the hypothesis that the primary defect caused by these genes results in decreased insulin secretion.

Type 2 diabetes is a very common metabolic disease and its incidence is increasing rapidly worldwide [1]. Despite five to six decades of extensive investigation, the basic physiologic defect responsible for type 2 diabetes mellitus (T2DM) is still not known. It is now well accepted that T2DM develops when the beta-cell is unable to supply the amount of insulin needed to maintain normal glucose levels [2]. But is the primary problem in the beta-cell itself? Or is the problem resistance to insulin action, which puts an abnormally high load on the beta-cell, causing it to eventually fail?

Epidemiologic studies have shown that genetic variation plays a very important part in determining the risk of developing diabetes. Currently available data suggest that for the vast majority of patients with T2DM, the genetic risk is determined by a variable combination of an unknown number of common genetic variants. Any of these variants alone is neither sufficient nor necessary for diabetes to occur. Genetic analysis of T2DM has yielded only a few such genetic variants that are consistently associated with risk of disease. The study of monogenic disorders of glucose metabolism may shed light on potential mechanisms for the common form of T2DM.

## Gene Mutations in Monogenic Diabetes

To date, mutations in more than ten genes have been associated with different forms of monogenic diabetes. Maturity-onset diabetes of the young (MODY), an autosomal dominant disease with onset of diabetes typically before the age of 25 years, can be caused by mutations in more than six genes [3]. Neonatal diabetes, either transient or permanent, can be caused by mutations in at least four different genes as well as an imprinting disorder on chromosome 6, the precise genetic mechanism of which has yet to be defined [4]. Common polymorphisms in some of these genes have been associated with increased risk of developing the more common form of polygenic T2DM. Most of the diabetes-related genes thus far identified are expressed in beta-cells and the diabetes-associated mutations are thought to result in decreased beta-cell function and inadequate insulin secretion. In an article published in *PLoS Medicine*, Pearson et al. report the birth weight of patients carrying a mutation in either of two closely related genes associated with the MODY syndrome, *HNF4A* and *HNF1A*, testing the hypothesis that the primary defect caused by these genes results in decreased insulin secretion [5].

## The New Study

Birth weight is largely determined by fetal insulin secretion. Defective insulin secretion, such as is found in patients with neonatal diabetes, is associated with low birth weight [6]. In contrast, beta-cell defects that cause increased, unregulated insulin secretion as is found in hyperinsulinemic hypoglycemia of infancy are associated with macrosomia [7]. In addition, maternal factors influence fetal insulin secretion, and thus fetal growth, independently of specific genetic defects. Infants of diabetic mothers are exposed to increased glucose levels, which trigger increased fetal insulin secretion and macrosomia. Maternal malnutrition or placental insufficiency results in decreased nutrient supply to the fetus, decreased fetal insulin secretion, and poor fetal growth. Interestingly, inappropriately low birth weight has been shown to be an important and highly significant predictor of late-onset T2DM [8,9].

Since both *HNF1A* and *HNF4A* are associated with insulinopenic diabetes in young adults [10,11], it would be expected that affected newborns would be either small for gestational age, if the insulin secretion defect is evident during fetal life, or normal for gestational age, if the functional defect develops after birth. Furthermore, both *HNF1A* and *HNF4A* are thought to interact with each other, affecting each other's expression level in the beta-cell [12–14]. Patients with diabetes caused by *HNF1A* and *HNF4A* mutations are phenotypically similar. It was expected, therefore, that defects in either gene would affect fetal growth in the same way.

Surprisingly, Pearson et al. found that *HNF4A* mutations were associated with increased fetal weight in patients, whereas *HNF1A* mutations did not appear to affect fetal weight at all. This increase in fetal growth found in *HNF4A* mutation carriers appears to be a direct result of the fetal genotype and is not significantly altered by maternal genotype. Similar weight gain was seen in fetuses carrying an *HNF4A* mutation, regardless of whether the mutation was inherited from the father or the mother. Furthermore, this mutation-associated increase in birth weight is also seen when genetically discordant siblings are compared, negating the importance of intrauterine environment in determining fetal weight in this setting.

To determine if this increase in birth weight is indeed caused by fetal hyperinsulinism, Pearson et al. reviewed patient records, and determined that eight out of 54 of the patients with *HNF4A* mutations had transient neonatal hypoglycemia, and in three of these elevated insulin levels were documented. In contrast, none of the normal siblings or the patients carrying an *HNF1A* gene mutation had neonatal hypoglycemia. This unexpected finding was confirmed in a mouse model of beta-cell-specific *HNF4A* depletion, which resulted in intrauterine and postnatal hyperinsulinism, with hypoglycemia in the postnatal period.

## Implications of the Findings

Thus, it appears that MODY-associated *HNF4A* mutations cause increased insulin secretion in the fetal and neonatal period, resulting in increased birth weight and neonatal hypoglycemia. Over time, the beta-cell function decreases, and insulinopenic diabetes develops, usually during the third decade of life.

This is not the first example of fetal hyperinsulinism developing into insulinopenic diabetes later in life. Huopio et al. report that dominant inactivating mutations in the sulfonylurea receptor (*ABCC8*), a major component of the beta-cell ATP-regulated potassium channel, result in a similar phenotype: hyperinsulinemic hypoglycemia with macrosomia in infancy, decreased glucose-stimulated insulin secretion in adolescence and young adulthood, and frank diabetes during middle age [15,16]. The mechanism by which this occurs is not known, but it was proposed that chronic depolarization of the beta-cell plasma membrane results in abnormal opening of voltage-gated potassium channels, causing elevated intracellular calcium, which triggers apoptosis pathways. To determine if *HNF4A* mutations recapitulate this series of events by modulating the expression of either of the two genes that make up the K_ATP_ channel (*ABCC8* and *KCNJ11*), Pearson et al. determined the expression of these genes in islets isolated from their mutant mice, but found no evidence of abnormal expression levels.

How then does this relate to the pathogenesis of polygenic T2DM? To define the mechanism by which genetic variation predisposes to T2DM, two major scenarios have been proposed. In the first scenario, decreased beta-cell function, due to a primary beta-cell defect, predisposes to diabetes. In the presence of increased demand for insulin, such as obesity-related insulin resistance or physical inactivity, this relative inability to increase insulin production becomes more evident and the incidence of frank diabetes increases. In addition, it has been hypothesized that fetal nutritional deprivation “programs” the beta-cells so that they cannot adequately compensate for increased insulin demands later in life, providing an environmental mechanism with the same end result.

In the second scenario, there is a primary defect causing insulin resistance. However, only a fraction of patients with insulin resistance will develop frank diabetes. In the majority, the pancreas will compensate with increased insulin secretion, maintaining euglycemia. Thus, a second “defect” limiting the pancreas's ability to compensate for insulin resistance is necessary for diabetes to occur. However, this may not be a distinct defect per se, but may simply represent the lower end of a normally distributed ability to increase beta-cell mass or function in response to increased demand.

Genetic data that have accumulated over the last ten years suggest that T2DM is a genetically heterogeneous disease in which either or both of these two scenarios could be important in any given individual. The current study demonstrates that a third scenario, in which a primary beta-cell defect results in increased insulin secretion, and thus increased fetal growth, long-term beta-cell damage, and late-onset insulin deficiency, may also be important in the pathogenesis of diabetes in some patients. It is not known how the same *HNF4A* mutation causes hyperinsulinism in the fetus and hypoinsulinism in the adult. Nor is it known why two very closely related genes, *HNF4A* and *HNF1A*, cause such different phenotypes. Genetic variants in the beta-cell specific (P2) promoter of *HNF4A* have been associated with polygenic T2DM. Could the scenario described by Pearson et al. be a common pathogenic mechanism for T2DM? Future studies are likely to address these important questions.

## Abbreviations

MODY: maturity-onset diabetes of the young
T2DM: type 2 diabetes mellitus

## References

[pmed-0040148-b001] Zimmet P, Alberti KG, Shaw J (2001). Global and societal implications of the diabetes epidemic. Nature.

[pmed-0040148-b002] Weir GC, Laybutt DR, Kaneto H, Bonner-Weir S, Sharma A (2001). Beta-cell adaptation and decompensation during the progression of diabetes. Diabetes.

[pmed-0040148-b003] Vaxillaire M, Froguel P (2006). Genetic basis of maturity-onset diabetes of the young. Endocrinol Metab Clin North Am.

[pmed-0040148-b004] Diatloff-Zito C, Nicole A, Marcelin G, Labit H, Marquis E (2007). Genetic and epigenetic defects at the 6q24 imprinted locus in a cohort of 13 patients with transient neonatal diabetes: New hypothesis raised by the finding of a unique case with hemizygotic deletion in the critical region. J Med Genet.

[pmed-0040148-b005] Gloyn AL, Pearson ER, Antcliff JF, Proks P, Bruining GJ (2004). Activating mutations in the gene encoding the ATP-sensitive potassium-channel subunit Kir6.2 and permanent neonatal diabetes. N Engl J Med.

[pmed-0040148-b006] Pearson ER, Boj SF, Steele AM, Barrett T, Stals K (2007). Macrosomia and hyperinsulinaemic hypoglycaemia in patients with heterozygous mutations in the *HNF4A* gene. PLoS Med.

[pmed-0040148-b007] Glaser B (2000). Hyperinsulinism of the newborn. Semin Perinatol.

[pmed-0040148-b008] Hales CN, Barker DJ, Clark PM, Cox LJ, Fall C (1991). Fetal and infant growth and impaired glucose tolerance at age 64. BMJ.

[pmed-0040148-b009] Lawlor DA, Davey Smith G, Clark H, Leon DA (2006). The associations of birthweight, gestational age and childhood BMI with type 2 diabetes: Findings from the Aberdeen Children of the 1950s cohort. Diabetologia.

[pmed-0040148-b010] Lehto M, Tuomi T, Mahtani MM, Widen E, Forsblom C (1997). Characterization of the MODY3 phenotype. Early-onset diabetes caused by an insulin secretion defect. J Clin Invest.

[pmed-0040148-b011] Gupta RK, Vatamaniuk MZ, Lee CS, Flaschen RC, Fulmer JT (2005). The MODY1 gene *HNF-4alpha* regulates selected genes involved in insulin secretion. J Clin Invest.

[pmed-0040148-b012] Kuo CJ, Conley PB, Chen L, Sladek FM, Darnell JE (1992). A transcriptional hierarchy involved in mammalian cell-type specification. Nature.

[pmed-0040148-b013] Boj SF, Parrizas M, Maestro MA, Ferrer J (2001). A transcription factor regulatory circuit in differentiated pancreatic cells. Proc Natl Acad Sci U S A.

[pmed-0040148-b014] Thomas H, Jaschkowitz K, Bulman M, Frayling TM, Mitchell SM (2001). A distant upstream promoter of the *HNF-4alpha* gene connects the transcription factors involved in maturity-onset diabetes of the young. Hum Mol Genet.

[pmed-0040148-b015] Huopio H, Reimann F, Ashfield R, Komulainen J, Lenko HL (2000). Dominantly inherited hyperinsulinism caused by a mutation in the sulfonylurea receptor type 1. J Clin Invest.

[pmed-0040148-b016] Huopio H, Otonkoski T, Vauhkonen I, Reimann F, Ashcroft FM (2003). A new subtype of autosomal dominant diabetes attributable to a mutation in the gene for sulfonylurea receptor 1. Lancet.

